# Association of the American Heart Association’s new “Life’s Essential 8” with all-cause mortality in patients with chronic kidney disease: a cohort study from the NHANES 2009–2016

**DOI:** 10.1186/s12889-024-19138-w

**Published:** 2024-06-19

**Authors:** Yingdong Han, Hong Di, Yibo Wang, Yun Zhang, Xuejun Zeng

**Affiliations:** Department of Family Medicine & Division of General Internal Medicine, Department of Medicine. Peking Union Medical College Hospital, State Key Laboratory of Complex Severe and Rare Diseases (Peking Union Medical College Hospital), Chinese Academy of Medical Sciences, No. 1 Shuaifuyuan, Dongcheng District, Beijing, 100730 China

**Keywords:** Chronic kidney disease, Life’s essential 8, Cardiovascular health, NHANES, All-cause mortality

## Abstract

**Background:**

People with chronic kidney disease (CKD) are more likely to die prematurely, and this increased risk of death is primarily attributable to deaths from cardiovascular disease (CVD). We aim to investigate the relationship between Life’s Essential 8 (LE8), a newly proposed cardiovascular health (CVH) measurement system, and all-cause mortality of CKD patients among US adults.

**Methods:**

A total of 3,169 CKD patients aged 20 and older from the National Health and Nutritional Examination Survey in 2009–2016 were involved in this study. Participants were divided into low (0–49), moderate (50–79) and high (80–100) CVH groups according to LE8 score (range 0-100). The mortality was ascertained from the National Death Index. Cox proportional hazards regression and restricted cubic spline were used to investigate the relationship.

**Results:**

Among the 3,169 CKD patients, the median age was 66.0 (25.0) years and 1,671 (52.7%) were female, and the median follow-up time was 6.00 years. The median LE8 score of the study cohort was 57.5 (19.4). CKD patients with low CVH, health behavior (HB) and health factors (HF) scores presented with higher all-cause mortality (both log-rank *P*-values < 0.001). After adjusted for multiple confounders, patients in higher CVH group had a lower risk of all-cause mortality, with a HR (95%CI) of 0.32 (0.19–0.55). Similar results were observed in high HB group [HR 0.36 (0.25–0.50)]. The restricted cubic spline showed a significant inverse relationship between LE8, HB and HF scores with CKD all-cause mortality, while the protective effect seemed weaker for HF score. Above results remained robust in the sensitivity analysis. Stronger inverse associations were revealed in middle-aged patients and patients with higher education levels.

**Conclusions:**

LE8 and its subscales scores were inversely associated with all-cause mortality in patients with CKD. Promoting CVH in CKD patients is a potential way to improve their long-term survival rate.

**Supplementary Information:**

The online version contains supplementary material available at 10.1186/s12889-024-19138-w.

## Introduction

Chronic kidney disease (CKD) is defined as decreased kidney function shown by markers of kidney damage or glomerular filtration rate (GFR) of < 60 mL/min/1.73 m², or both, for more than 3 months, regardless of the underlying cause [1]. Although the estimates of CKD prevalence, incidence and progression vary globally, all epidemiological studies suggest substantial burden from CKD [2, 3]. Previous epidemiological studies estimated the global prevalence of CKD to be 3–18% and with a substantial contribution from the elderly population [4]. In addition, socioeconomic status also plays an important role in the incidence and prevalence of CKD [1]. In America, CKD affects more than 20 million people, and over 500,000 have end-stage renal disease (ESRD) [5]. On top of being a precursor to ESRD, CKD has been recognized as a vital risk factor for cardiovascular disease (CVD), cognitive dysfunction, and all-cause mortality [6]. Anemia, inflammation, volume overload, and uremic toxins are the pathophysiologic mechanisms that link CKD to the development of CVD [7]. People with CKD are more likely to die prematurely, and this increased risk of death rises exponentially with worsening renal function, primarily attributable to CVD [1]. 87% of adults aged 45 or older were diagnosed CVD at the time of ESRD onset, and approximately 50% of deaths were attributed to CVD [8]. Diabetes, hypertension and obesity are the main common risk factors of CKD and CVD in middle-income and high-income countries [9].

In 2010, the American Heart Association (AHA) proposed Life’s Simple 7 (LS7) to monitor and promote cardiovascular health (CVH), measured on the basis of 7 health behaviors and factors. Each metric is demonstrated as a ternary-ordered category (poor, intermediate and ideal) [10], and the prevalence of having ≥ 5 metrics at ideal levels is only 4% among adults ≥ 60 years of age, 11% among adults aged 40 to 59 years, 32% among adults 20 to 39 years of age, and 45% among US adolescents [11]. Previous studies have suggested inverse associations between the number of ideal CVH metrics and all-cause mortality and CVD mortality. A meta-analysis suggested that people having ≥ 5 ideal CVH metrics were associated with a relative risk of 0.25 for CVD mortality and 0.55 for all-cause mortality compared with 0 to 2 ideal CVH metrics [12].

Life’s Essential 8 (LE8) is an updated and modified version of LS7, consisting of four health behavior and four health factors, with sleep health as a new CVH component [10]. All 8 metric are quantified into a 100-point scale and higher points are thought to be healthier. Compared with the original score system, LE8 is more comprehensive and sensitive to account for intra-individual and inter-individual differences through describing CVH in a broader manner [10, 13].

Multifactorial intervention to mitigate the risk of CVD is necessary to improve the survival rate in CKD patients [1, 7]. Achieving a higher CVH score according to the LE8 score is associated with healthy longevity, and a lower risk of CVD mortality and all-cause mortality [13–15]. The Finnish men within LE8 top quartile had 60% lower risk of CVD mortality when compared with those within the bottom quartile [14]. Previous guidelines usually recommend controlling blood pressure, glucose and proteinuria, in combination with improving anemia to reduce the risk of CKD progression and mortality rates [16–18]. LE8 has not been applied to the field of CKD so far, and exploring the close and intertwined relationship between LE8 and CKD must be a good attempt to promote and reinforce healthy metrics and to improve life qualities and longevities.

Here, we investigate the association between LE8 score and all-cause mortality of CKD patients using National Health and Nutrition Examination Surveys (NHANES) data. The NHANES data include representative sampling across demographic groups and are identified as the best available source to monitor population-level CVH.

## Materials and methods

### Data source and study population

The NHANES, which collects the nutritional and health information of the US population every 2 years, is a periodic, cross-sectional health survey program using multistage, stratified sampling design to collect a nationally representative sample of non-institutionalized civilians. The survey consists of an interview and a physical examination administered by a trained medical worker as well as laboratory tests. The National Center for Health Statistics’ Research Ethics Review Board reviewed and approved all data collection protocols. Written informed consent was obtained before the interview and examination stages from all participants and all data were de-identified by the National Center for Health Statistics before being made publicly available [19]. The investigation conformed with the principles outlined in the Declaration of Helsinki.

The population for this research consisted of four consecutive cycles of NHANES from 2009 to 2016 (2009–2010, 2011–2012, 2013–2014 and 2015–2016). Among the 40,439 participants in the examination, we excluded those who were less than 20 years of age (*n* = 17,173). Among the 23,266 participants, we excluded those with incomplete information for all LE8 metrics and with ineligible data on mortality follow-up (*n* = 4,399). Finally, a total of 3,169 unweighted (Weighted *n* = 26,857,095) CKD patients were involved in this study (Additional Fig. 1), including 1,554 patients of CKD stages 3–5 and 1,371 patients of CKD stage 3.

### Measurement of LE8

LE8 scoring algorithm consists of 4 health factors (HF) (blood lipids, blood glucose, blood pressure and BMI) and 4 health behaviors (HB) (diet, physical activity, sleep health and nicotine exposure). Detailed algorithms for calculating the LE8 scores for each of the metrics to NHANES data have been previously published and can be found in Additional Table 1 [10, 20]. Each of the 8 CVH metrics was scored ranging from 0 to 100 points. The overall LE8 score was calculated as the average of all 8 CVH metrics. AHA recommends that participants with overall CVH scores of 80 to 100 be considered high CVH; 50–79, moderate CVH; and 0–49 points, low CVH [10].

Diet metric was evaluated by the Healthy Eating Index (HEI) 2015 [21]. The components and scoring standards of HEI-2015 were summarized in Additional Table 2. The dietary intakes of participants collected from two 24 h dietary recalls were combined with the United States Department of Agriculture food patterns equivalents data to construct and calculate the HEI-2015 scores [22]. The simple HEI scoring algorithm method (by person) was used to compute the HEI-2015 score using an official SAS code provided by National Cancer Institute [23].

Self-report questionnaires were employed to collect information about participants’ physical activity and sleeping information, nicotine exposure, diabetes history and medication history [24]. Height, weights and blood pressure were measured, and BMI was calculated by weight and height. Data on blood lipids, plasma glucose, hemoglobin A1c, uric acid, creatinine and urine albumin creatinine ratio (UACR) were also collected.

### Study variables

A definition of CKD included persons with an eGFR ≤ 60 ml/min/1.73m^2^ or a one-time UACR ≥ 30 mg/g. The eGFR was calculated according to the CKD-Epidemiology collaboration equation [6, 25].

Potential confounding factors in this study include age, race/ethnicity (Mexican American, Other Hispanic, Non-Hispanic White, Non-Hispanic Black, and Other race), gender (Male, Female), education levels (High school graduate or less, Some college or AA degree, and College graduate or above), marital status (Coupled, Single or separated), poverty ratio (< 1.0, ≥ 1.0), obesity status (Normal: <25 kg/m^2^, Overweight: 25–30 kg/m^2^, Obesity: >30 kg/m^2^) and uric acid.

### Mortality ascertainment

National Center for Health Statistics has linked data from various surveys with death certificate records from the National Death Index with follow-up through NHANES 1999–2018. The Linked Mortality Files have been updated with mortality follow-up data through December 31, 2019 [26]. The underlying cause of death was determined by the ICD-10. All-cause mortality was defined as any reason for death. The duration of follow-up was defined as the interval from the interview date to the date of death or through December 31, 2019 for participants without event.

### Statistical analysis

To account for the complex sampling design and ensure nationally representative estimates, all analyses were adjusted for survey design and weighting variables. New sample weight (the original 2-year sample weight divided by 4) was constructed according to the analytical guidelines of the NHANES [27]. Continuous variables were described with median (interquartile range), and categorical variables were reported as numbers (percentage). The median values among different CVH groups (Low CVH: 0–49; Moderate CVH: 50–79; High CVH: 80–100) were compared with the Kruskal Wallis test. The Chi-square test was adopted to compare the percentages of categorical variables among different CVH groups. The Bonferroni test was used for the intergroup comparison. We used the same definition and cut-off points to categorize HB and HF scores to further investigate the association between LE8 and its subscales scores and the all-cause mortality of CKD patients in this study. A direct method of standardization was used to calculate the crude rates of all-cause mortality per 1000 person-years and age- and sex- adjusted rates of all-cause mortality per 1000 person-years. The overall survival time was illustrated by a Kaplan-Meier curve, and group differences were examined using the log-rank test. Kaplan-Meier cumulative incidence curves were also generated for the calculation of cumulative mortality using three categories of CVH metrics (low, moderate and high).

Survey-weighted multivariable Cox proportional hazards regression models were used to calculate hazard ratios (HRs) and 95%CIs for the associations of LE8 scores with risk of all-cause mortality after the adjustment of potential confounders. Schoenfeld residuals were used to test the proportional hazards assumption, and no violation was observed. In model 1, we adjusted for age, gender, race, and obesity status. In model 2, poverty status, education levels, marital status and uric acid were additionally adjusted. Restricted cubic spline analysis was applied to characterize the dose-response relationship between the LE8 score and its subscales score with all-cause mortality in patients with CKD. Nonlinearity was tested using the likelihood ratio test.

We further performed stratified analyses by gender, age groups (20–45, 46–65, 66 and over), education levels (High school graduate or less, Some college or AA degree, and College graduate or above), poverty ratio (< 1.0, ≥ 1.0), marital status (Coupled, Single or separated), and obesity status (Normal, Overweight and Obesity). The *P* values for the production terms between LE8 scores and the stratified factors were used to estimate the significance of interactions.

To assess the robustness of the results, we performed the following sensitivity analyses: (1) dividing the LE8 scores, HB and HF scores into 4 groups according to the quartiles, with the first quartile (Q1 group) as the reference group; (2) using propensity score matching to correct the confounding factors (age, sex, race, uric acid, obesity status, poverty status, education levels, and marital status) between the survival group and mortality group; (3) excluding CKD (stages 1–2) patients and repeating the main analyses.

Statistical tests were 2-sided, and statistical significance was set at *P* < 0.05. All analyses were performed with SPSS 23.0 (IBM Corporation, Chicago, USA) SAS version 9.4 (SAS institute, Cary, NC) and R 4.1.0. (Core Team, Vienna, Austria).

## Results

### Baseline characteristics

A total of 3,169 CKD patients aged 20 and older were included. Baseline characteristics of the study population were summarized by the category of CVH status in Additional Table 3. The median age of the patients was 66.0 (25.0) years, and 1671 (52.7%) were women. The median LE8 score for all participants, low, moderate and high CVH group were 57.5 (19.4), 42.5 (8.8), 61.9 (13.1) and 85.0 (6.9), respectively. The median of HB score and HF score for the participants were 62.5 (26.3) and 53.8 (26.3). Patients in the high CVH group were younger and had higher educational level and eGFR level, while patients in the low CVH group were more likely to be obese and had higher creatinine, UACR and uric acid level. The proportion of CKD patients (stages 3, stages 4–5 and stages 3–5) was significantly lower in high CVH group compared with low and moderate CVH groups.

### Survival analysis

The survival status of participants according to the CVH metrics was presented in Table 1. The unweighted total cases/participants were 792/3169 and the weighted total cases/participants were 5,834,743/26,857,095. The crude all-cause mortality and age- and sex-standardized all-cause mortality per 1000 person-years among adults with total CVH scores at the moderate [Crude: 38.42 (35.17, 41.94); Adjusted: 33.29 (30.27, 36.59)] and high [Crude: 16.11 (10.67, 24.05); Adjusted: 14.96 (9.75, 22.70)] levels were significantly lower than those with low [Crude: 51.27 (45.63, 57.54); Adjusted: 44.04 (38.81, 49.91)] level.

**Table 1:** Survival status of participants according to the total scores of cardiovascular health metrics

	Low CVH group	Moderate CVH group	High CVH group
Cases/participants (Unweighted)	279/898	488/2042	25/229
Cases/participants (Weighted)	1,937,883/6,551,503	3,716,558/17,473,023	180,302/2,832,569
Crude mortality rate per 1000 person-years	51.27 (45.63, 57.54)	38.42 (35.17, 41.94)	16.11 (10.67, 24.05)
Age- and sex-standardized all-cause mortality rate per 1000 person-years	44.04 (38.81, 49.91)	33.29 (30.27, 36.59)	14.96 (9.75, 22.70)

**Fig. 1:**
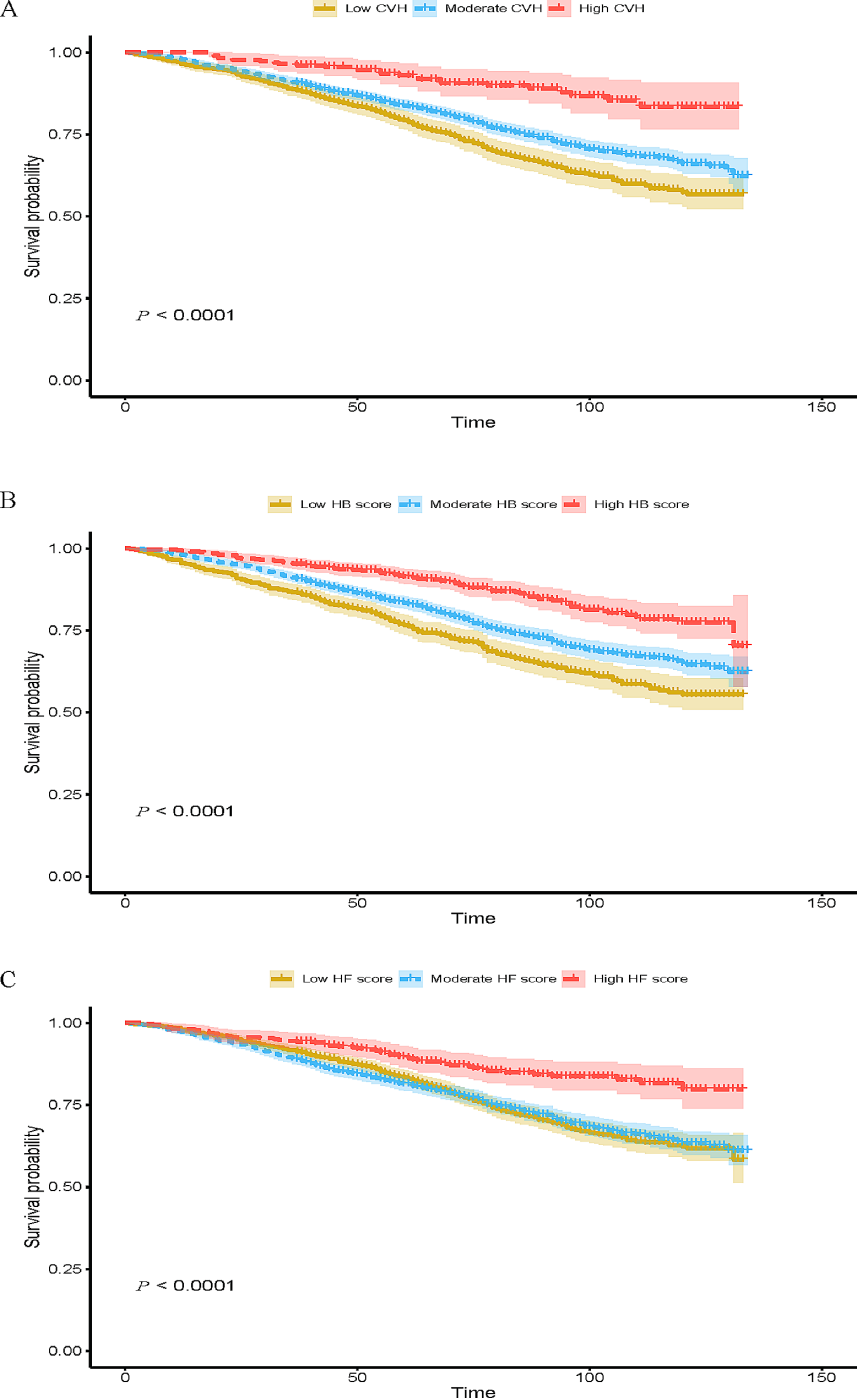
Kaplan-Meier survival curves for all-cause mortality in chronic kidney disease patients according to CVH status (**A**), Health Behavior score (**B**) and Health Factors Score (**C**)

**Fig. 2:**
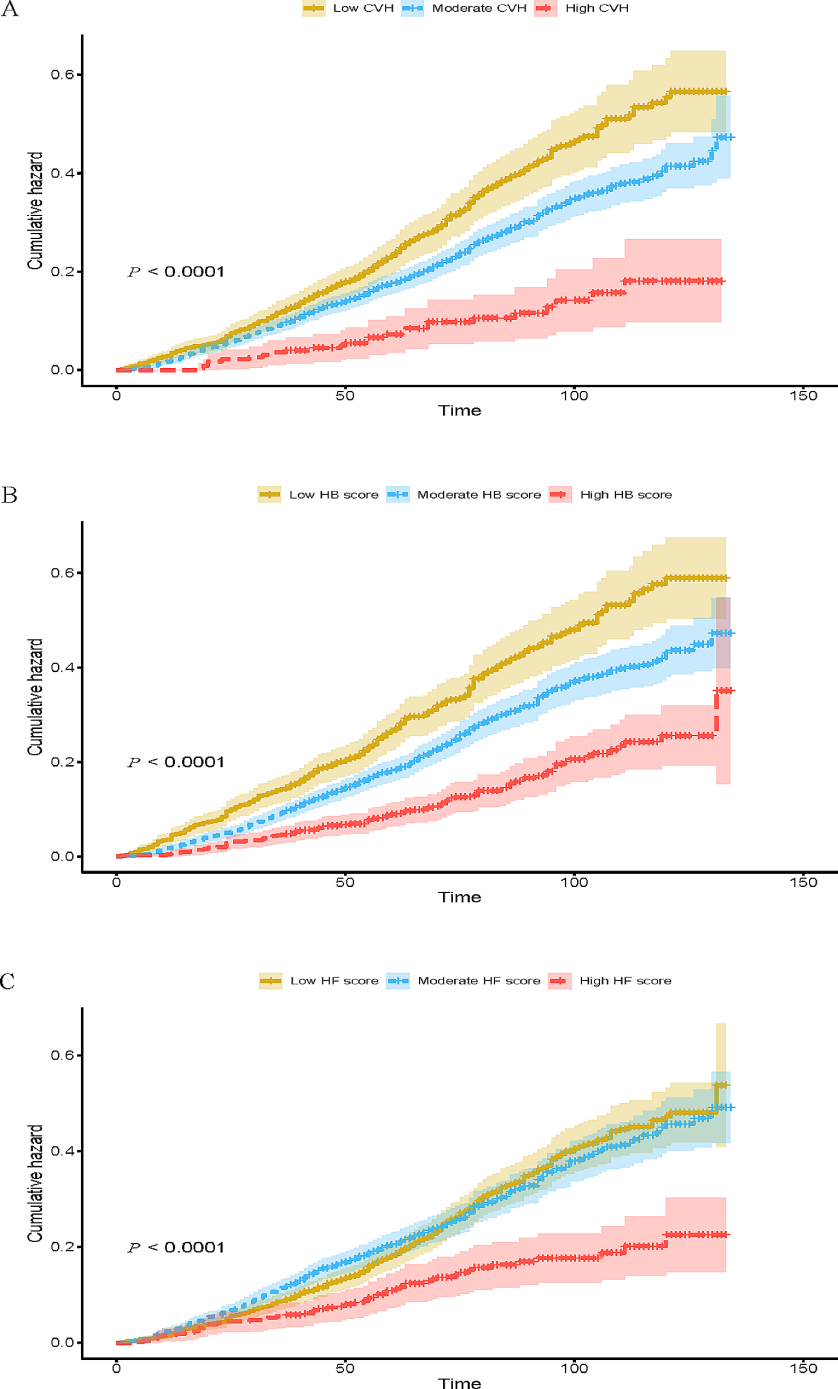
Kaplan-Meier cumulative incidence curves for all-cause mortality in chronic kidney disease patients according to CVH status (**A**), Health Behavior score (**B**) and Health Factors Score (**C**)

The Kaplan-Meier survival curves for all-cause mortality of CKD patients are displayed in Fig. 1. The median follow-up time was 6.00 years. The 10-year survival rates were 79.3%, 83.8% and 93.0% for CKD patients in the low, moderate and high CVH groups, respectively. Compared to those in the moderate and high groups, CKD patients in the low CVH group and HB group displayed higher all-cause mortality (both log-rank *P*-values < 0.001). CKD patients in the high HF group displayed lower all-cause mortality compared with those in low and moderate HF groups. The Kaplan-Meier cumulative incidence curves of CKD patients are displayed in Fig. 2. CKD patients who achieved a higher CVH score had a significantly lower cumulative incidence rate of all-cause mortality (log-rank *P*-values < 0.001).

### LE8 score and all-cause mortality in CKD patients

The associations between LE8 and all-cause mortality of CKD patients are displayed in Table 2. After adjusting for age, gender, race, and obesity status, the HRs (95% CI) of all-cause mortality were 0.57 (0.48–0.67) in the moderate CVH group and 0.23 (0.14–0.39) in the high CVH group, respectively. In the fully adjusted model (model 2), the HRs of all-cause mortality were 0.66 (0.56–0.77) in the moderate CVH group, and 0.32 (0.19–0.55) in the high CVH group, respectively.

**Table 2:** Survey-weighted association of Life’s Essential 8 score with all-cause mortality of chronic kidney disease patients

	Crude model		Multivariable model 1		Multivariable model 2	
	HR (95% CI)	*P* value	HR (95% CI)	*P* value	HR (95% CI)	*P* value
LE8 score						
Low (0–49)	1 (Reference)	/	1 (Reference)	/	1 (Reference)	/
Moderate (50–79)	0.69 (0.59–0.81)	< 0.01	0.57 (0.48–0.67)	< 0.01	0.66 (0.56–0.77)	< 0.01
High (80–100)	0.19 (0.11–0.33)	< 0.01	0.23 (0.14–0.39)	< 0.01	0.32 (0.19–0.55)	< 0.01
Per 10 points increase	0.78 (0.74–0.82)	< 0.01	0.72 (0.67–0.76)	< 0.01	0.76 (0.71–0.81)	< 0.01
Health behaviors score						
Low (0–49)	1 (Reference)	/	1 (Reference)	/	1 (Reference)	/
Moderate (50–79)	0.74 (0.61–0.90)	< 0.01	0.69 (0.58–0.83)	< 0.01	0.76 (0.63–0.93)	< 0.01
High (80–100)	0.31 (0.22–0.45)	< 0.01	0.29 (0.21–0.41)	< 0.01	0.36 (0.25–0.50)	< 0.01
Per 10 points increase	0.84 (0.80–0.88)	< 0.01	0.81 (0.78–0.85)	< 0.01	0.85 (0.81–0.88)	< 0.01
Health factors score						
Low (0–49)	1 (Reference)	/	1 (Reference)	/	1 (Reference)	/
Moderate (50–79)	0.84 (0.71–0.99)	0.03	0.72 (0.62–0.85)	< 0.01	0.75 (0.63–0.90)	< 0.01
High (80–100)	0.37 (0.26–0.53)	< 0.01	0.60 (0.42–0.87)	< 0.01	0.64 (0.44–0.94)	0.02
Per 10 points increase	0.90 (0.86–0.93)	< 0.01	0.90 (0.85–0.94)	0.01	0.91 (0.87–0.96)	< 0.01

HR hazard ratio, CI confidence interval, LE8 life’s essential 8

Model 1 adjusted for age, sex, race, and obesity status

Model 2 additionally adjusted for uric acid, poverty status, education levels, and marital status

**Fig. 3:**
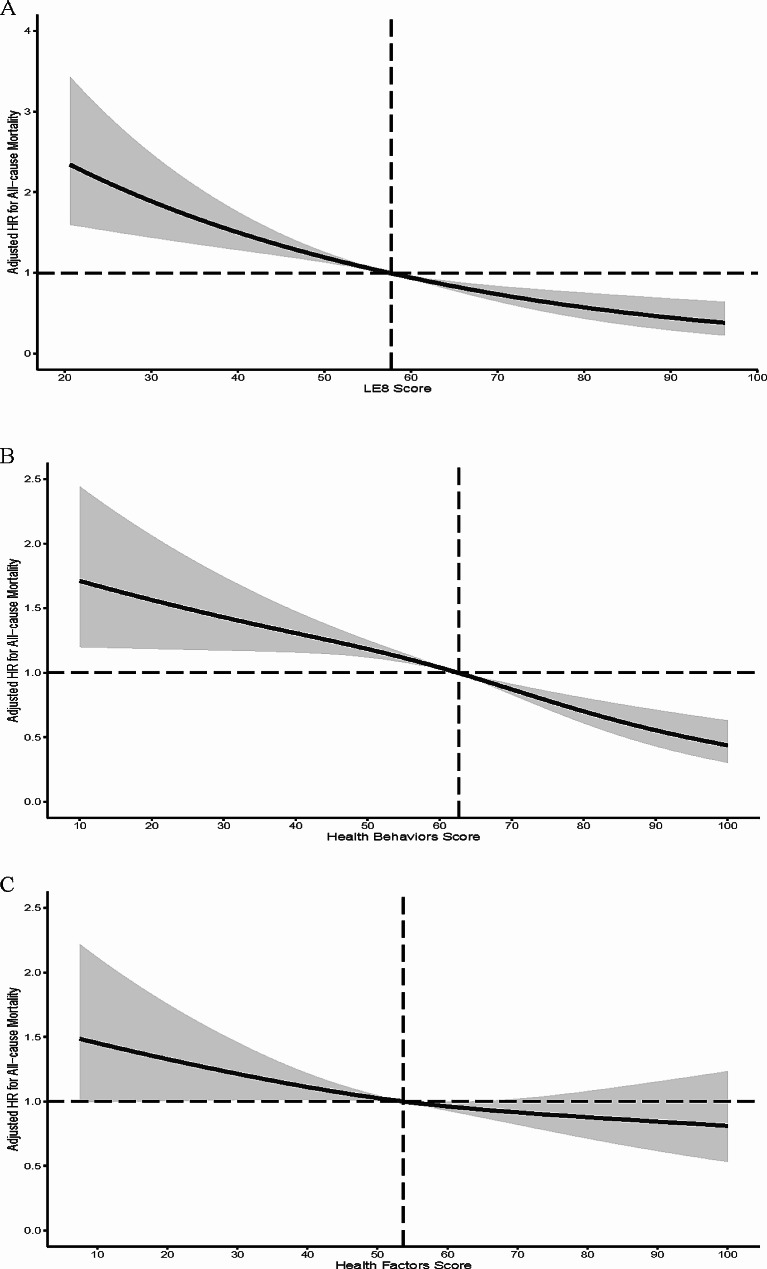
Dose-response relationships between Life’s Essential 8 scores (**A**), Health Behavior score (**B**), Health Factors Score (**C**), and all-cause mortality of chronic kidney disease patients. HRs (solid lines) and 95% confidence levels (shaded areas) were adjusted for age, sex, race/ethnicity, obesity status, uric acid, poverty status (as a binary variable), education level and marital status. Vertical dotted lines indicate the minimal threshold for the beneficial association with estimated HR = 1

In model 2, the HR for every 10-point increment in LE8 score was 0.76 (0.71–0.81) in association with CKD all-cause mortality. An inverse association was observed between the LE8 score and all-cause mortality (*P* < 0.01; Fig. 3A). The minimal threshold for the beneficial association was 57.6 scores (estimate HR = 1).

### Health behavior scores and all-cause mortality in CKD patients

In model 1, the HRs (95%CI) of all-cause mortality were 0.69 (0.58–0.83) and 0.29 (0.21–0.41) in the moderate and high HB groups, respectively compared with the low HB group. In the fully adjusted model 2, the HRs of the moderate and high HB groups were 0.76 (0.63–0.93) and 0.36 (0.25–0.50), respectively compared with the low HB group.

In model 2, the HR for every 10-point increment in HB score in association with the all-cause mortality of CKD patients was 0.85 (0.81–0.88). Restricted cubic spline analysis (Fig. 3B) revealed that the HB score was negatively correlated with all-cause mortality in CKD patients (*P* < 0.01). The minimal threshold for the beneficial association was 62.7 (estimate HR = 1).

### Health factor score and all-cause mortality in CKD patients

The HRs of CKD all-cause mortality was 0.75 (0.63–0.90) for the moderate HF group and 0.64 (0.44–0.94) for the high HF group, compared to the low HF group in model 2. The HR for every 10-point increment in HF score was 0.91 (0.87–0.96) in association with CKD all-cause mortality. Restricted cubic spline analysis (Fig. 3C) revealed that the HF score was negatively correlated with all-cause mortality in CKD patients (*P* = 0.03). The minimal threshold for the beneficial association was 53.7 scores (estimate HR = 1).

### Subgroup analysis

The results of subgroup analysis are shown in Additional Fig. 2. The LE8 score (per 10 points increase) was negatively associated with all-cause mortality of CKD patients in almost all subgroups except for patients aged 20–45. Significant interaction was observed between LE8 with age and education level. Stronger inverse associations were observed in middle-aged patients (HR for per 10 scores increase, 0.66 (0.52–0.84)), patients with higher education levels (HR for per 10 scores increase, 0.67 (0.58–0.77), 0.64 (0.51–0.79)).

### Sensitivity analysis

The results above were proved robust in the sensitivity analysis. The sensitivity analysis was conducted for the association of LE8 scores and all-cause mortality of CKD patients according to quartiles of LE8 scores (Table 3). CKD patients in the highest LE8 groups, HB groups and HF groups were still significantly associated with lower all-cause mortality in model 2 and the protective effect of HF scores appeared weaker.

**Table 3:** Sensitivity analysis for the association of Life’s Essential 8 score with all-cause mortality of chronic kidney disease patients according to quartiles of Life’s Essential 8 score

	Crude model		Multivariable model 1		Multivariable model 2	
	HR (95% CI)	*P* value	HR (95% CI)	*P* value	HR (95% CI)	*P* value
LE8 score						
Q1	1 (Reference)	/	1 (Reference)		1 (Reference)	
Q2	0.85 (0.70–1.01)	0.07	0.72 (0.57–0.90)	< 0.01	0.79 (0.63–0.99)	0.04
Q3	0.74 (0.58–0.95)	0.02	0.54 (0.43–0.68)	< 0.01	0.62 (0.49–0.77)	< 0.01
Q4	0.36 (0.28–0.46)	< 0.01	0.32 (0.25–0.41)	< 0.01	0.40 (0.31–0.52)	< 0.01
Health behaviors score						
Q1	1 (Reference)	/	1 (Reference)	/	1 (Reference)	/
Q2	0.81 (0.63–1.04)	0.09	0.73 (0.58–0.93)	0.01	0.76 (0.60–0.97)	0.03
Q3	0.67 (0.53–0.85)	< 0.01	0.63 (0.51–0.79)	< 0.01	0.71 (0.57–0.90)	< 0.01
Q4	0.32 (0.23–0.45)	< 0.01	0.29 (0.21–0.38)	< 0.01	0.33 (0.24–0.45)	< 0.01
Health factors score						
Q1	1 (Reference)	/	1 (Reference)	/	1 (Reference)	/
Q2	1.00 (0.83–1.21)	0.99	0.80 (0.66–0.98)	0.03	0.89 (0.73–1.09)	0.25
Q3	0.93 (0.73–1.19)	0.58	0.75 (0.60–0.93)	< 0.01	0.79 (0.64–0.98)	0.03
Q4	0.54 (0.43–0.69)	0.01	0.60 (0.45–0.79)	< 0.01	0.69 (0.52–0.92)	0.01

HR Hazard ratio, CI confidence interval, LE8 life’s essential 8

Model 1 adjusted for age, sex, race, and obesity status

Model 2 additionally adjusted for uric acid, poverty status, education levels and marital status

The distribution of characteristic and propensity scores of the matching study population was summarized in Additional Table 4 and Additional Fig. 3. The association of LE8 score and HB score remained strong after propensity score matching (Table 4, HR for per 10 points increase, 0.82 (0.76–0.88) and 0.86 (0.82–0.90)), while the protective effect of HF scores appeared weaker (HR for per 10 points increase, 0.94 (0.89–0.99)).

**Table 4:** Sensitivity analysis of the association of the Life’s Essential 8 scores with mortality of chronic kidney disease patients

	Propensity score matching*		CKD G3-G5^†^ (*n* = 1554)		CKD G3^†^ (*n* = 1371)	
	HR (95% CI)	*P* value	HR (95% CI)	*P* value	HR (95% CI)	*P* value
LE8 score						
Low (0–49)	1 (Reference)	/	1 (Reference)	/	1 (Reference)	/
Moderate (50–79)	0.63 (0.53–0.78)	< 0.01	0.61 (0.47–0.79)	< 0.01	0.65 (0.49–0.87)	< 0.01
High (80–100)	0.36 (0.20–0.65)	< 0.01	0.37 (0.20–0.68)	< 0.01	0.38 (0.20–0.72)	< 0.01
Per 10 points increase	0.82 (0.76–0.88)	< 0.01	0.75 (0.69–0.82)	< 0.01	0.78 (0.71–0.87)	< 0.01
Health behaviors score						
Low (0–49)	1 (Reference)	/	1 (Reference)	/	1 (Reference)	/
Moderate (50–79)	0.77 (0.63–0.95)	0.01	0.71 (0.55–0.92)	< 0.01	0.74 (0.56–0.99)	0.04
High (80–100)	0.40 (0.27–0.59)	< 0.01	0.39 (0.27–0.56)	< 0.01	0.42 (0.28–0.63)	< 0.01
Per 10 points increase	0.86 (0.82–0.90)	< 0.01	0.84 (0.79–0.89)	< 0.01	0.85 (0.80–0.91)	< 0.01
Health factors score						
Low (0–49)	1 (Reference)	/	1 (Reference)	/	1 (Reference)	/
Moderate (50–79)	0.87 (0.73–1.05)	0.14	0.74 (0.58–0.95)	0.02	0.78 (0.58–1.04)	0.08
High (80–100)	0.69 (0.49–0.96)	0.03	0.78 (0.52–1.16)	0.22	0.79 (0.50–1.24)	0.31
Per 10 points increase	0.94 (0.89–0.99)	0.02	0.92 (0.86–0.98)	0.01	0.93 (0.86–1.02)	0.12

HR hazard ratio, CI confidence interval, LE8 life’s essential 8

* Matching for age, sex, race, obesity status, uric acid, education levels, poverty status and marital status

^†^ Adjusted for age, sex, race, and obesity status, uric acid, education levels, poverty status and marital status

The results of sensitivity analysis after excluding CKD (stages 1–2) patients were summarized in Table 4. In patients with CKD stages 3–5 and when compared with the low CVH and HB groups, the HRs of all-cause mortality were 0.37 (0.20–0.68) and 0.39 (0.27–0.56) in the high CVH group and the high HB group, respectively. HR for every 10 points increase in LE8 and HB scores were 0.75 (0.69–0.82) and 0.84 (0.79–0.89) in association with CKD all-cause mortality, while the protective effect of HF score appeared weaker (HR for per 10 points increase, 0.92 (0.86–0.98)). In patients with stage 3 CKD and when compared with the low CVH and HB group, the HRs of all-cause mortality were 0.38 (0.20–0.72) and 0.42 (0.28–0.63) in the high CVH group and HB group, respectively. HR for every 10 points increase in LE8 and HB scores were 0.78 (0.71–0.87) and 0.85 (0.80–0.91) in association with CKD all-cause mortality, while the protective effect of HF score became insignificant (HR for per 10 points increase, 0.93 (0.86–1.02)).

## Discussion

To our knowledge, this is the first research to explore the relationship between LE8 and all-cause mortality in patients with CKD. Using a nationally representative cohort of US CKD patients, we found inverse correlation between LE8 score, HB and HF score with the all-cause mortality, while the protective effect of HF score appeared weaker. We illustrated the dose-response relationship and survival curves between LE8 score and its subscales with all-cause mortality in CKD patients. Robustness of the results was strengthened by sensitivity analysis. We found stronger inverse correlation in middle-aged patients and patients with higher education levels. These findings suggest that improving LE8 scores might lower the all-cause mortality of CKD patients.

We used a nationally representative cohort of US adult CKD patients, which increased the statistical power to provide reliable results. The unweighted estimates can significantly deviate from the properly-weighted estimates. We utilized the NHANES weights to account for the complex survey design and to combine estimates from different subgroups for obtaining national estimates that accurately reflect the true relative proportions of these groups in the U.S. population. Restricted cubic spline analyses characterized the dose-response relationship between CVH and all-cause mortality of CKD patients and identified the minimal threshold for the beneficial association (57.6 scores for LE8, 62.7 scores for HB and 53.7 scores for HF score). We found that that each 10-point increase in the LE8 score and HB score was associated with a 24% and 15% reduction, respectively, in the risk of all-cause mortality among CKD patients. In contrast, the reduction associated with the HF score was comparatively weaker at 9%, suggesting that more stringent criteria for HB score might be advantageous. The median follow-up time was long enough to demonstrate a complete tendency and the cohort study design strengthened the level of the evidence. Causal association might be inferred through the time sequence of LE8 and the death events in CKD patients. Multiple statistical analyses were used to prove the robustness of the results, such as fully adjusting confounding factors, conducting sensitivity analyses, subgroup analysis and propensity score matching. Thus, our results were convincing.

The LS7 and LE8 were initially proposed mainly for monitoring and promoting CVH. Maintaining optimal CVH behaviors was associated with nearly 50% lower risk for coronary events among people at high genetic risk, and this association underscored the significance of CVH behaviors [28, 29]. A previous study conducted by G. Magnussen, which involved 19,951 US adults aged 30–79 years, found that participants with high CVH scores had 58% reduced risk of all-cause mortality and 64% reduced risk of CVD-specific mortality compared with adults with low CVH scores [13]. A prospective study that involved 250,825 participants from UK Biobank found that participants in the lowest quartile of LE8 score had 2.07 times higher risk for major adverse cardiovascular events, compared with individuals in the highest quartile [30].

Previously studies employed LS7 to assess the risk and all-cause mortality of CKD. In the Atherosclerosis Risk in Communities cohort study, Casey M, et al. found a higher LS7 score was significantly associated with lower risk of CKD [31]. The REGARDS study with 3093 CKD patients found that having 5 or more ideal LS7 metrics was connected with a 30% decreased risk of all-cause mortality [32]. Our findings were consistent with previous studies, as we found an inverse association between LE8 and its subscale scores with all-cause mortality in CKD patients. The definition of CVH for each LS7 component was categorized into ideal, moderate, and poor CVH. However, this measurement could not be applied to evaluate dose-response effects. The updated LE8 score quantify the original metrics from LS7 in more detail, offering a more accurate representation of the entire range of CVH.

Studies have confirmed the relationship between LE8 scores and CVD mortality, and excess deaths of CKD patients are closely connected with CVD [10, 15, 20]. Therefore, it is meaningful to explore the association between LE8 and all-cause mortality in CKD patients. The underlying correlations between LE8 and CVD are mainly bridged by inflammation modulation, endothelial function, oxidative stress, and epigenetics [10]. Certain environmental, lifestyle and social factors, such as physical inactivity, poor diet and psychological stress and sleep disturbance can promote systemic chronic inflammation [33]. Persistent systemic chronic inflammation increases the risk of type 2 diabetes, metabolic syndrome, and CVD, which in turn impairs health status and contributes to premature mortality in CKD patients [33–35]. These findings provide valid biological evidence for the significance of improving CKD patients’ overall health and all-cause mortality.

There are several limitations that deserve mentioning. The sample size is relatively small due to the intrinsic limitation and selectivity of the NHANES population. Although we have fully adjusted the potential covariates, it was difficult to include all potential confounding factors. LE8 was only measured at the baseline. The dynamic changes in LE8 were not available because NHANES database did not provide follow-up examination, leaving us unable to dynamically evaluate longitudinal changes of patients’ CVH status. While a consistent inverse relationship between LE8 and the all-cause mortality in patients with CKD was observed in nearly all subgroups, heterogeneity between different subgroups existed, hindering the overall representativity of each subgroup. The HB metrics assessments were based on self-reported questionnaires, which might cause recalling errors and measurement inconsistencies due to individual interpretations of the metrics.

## Conclusion

In this nationally representative cohort study of US CKD patients, higher LE8 score and its subscale score were found to be associated with lower risk of all-cause mortality. The strength of the association between LE8 score and all-cause mortality in CKD patients differed within the study population. Our research indicates potential benefits of improving LE8 scores as an applicable and effective approach for promoting the overall health of CKD patients.

### Electronic supplementary material

Below is the link to the electronic supplementary material.

Supplementary Material 1

## Data Availability

The datasets supporting the conclusions of this article was available in the public repository as described below. National Health and Nutrition Examination Survey data are available from the National Center for Health Statistics (https://www.cdc.gov/nchs/nhanes/).

## Abbreviations

AHA: American Heart Association;
BMI: Body mass index;
CKD: Chronic kidney disease;
CVD: Cardiovascular disease;
CVH: Cardiovascular health;
ESRD: End-stage renal disease;
GFR: Glomerular filtration rate;
HB: Health behavior;
HEI: Healthy Eating Index;
HF: Health factor;
HR: Hazard ratio;
LE8: Life’s Essential 8;
LS7: Life’s Simple 7;
NAFLD: Non-alcoholic fatty liver diseases;
NHANES: National Health and Nutritional Examination Survey;
UACR: Urine albumin creatinine ratio
