# Supplementary material for: Association of the American Heart Association’s new “Life’s Essential 8” with all-cause mortality in patients with chronic kidney disease: a cohort study from the NHANES 2009–2016
Source: BMC Public Health. 2024 Jun 19;24:1637. doi: 10.1186/s12889-024-19138-w (PMC11186270; doi:10.1186/s12889-024-19138-w)
Supplement: Supplementary file 1 — Supplementary Material 1 [file 12889_2024_19138_MOESM1_ESM.docx]

**Additional file**

**Association of the American Heart Association's new “Life’s Essential 8” with all-cause mortality in patients with chronic kidney disease: a cohort study from the NHANES 2009-2016.**

**Table of Contents**

Additional Figure 12

Additional Table 13

Additional Table 26

Additional Table 38

Additional Figure 210

Additional Table 411

Additional Figure 312


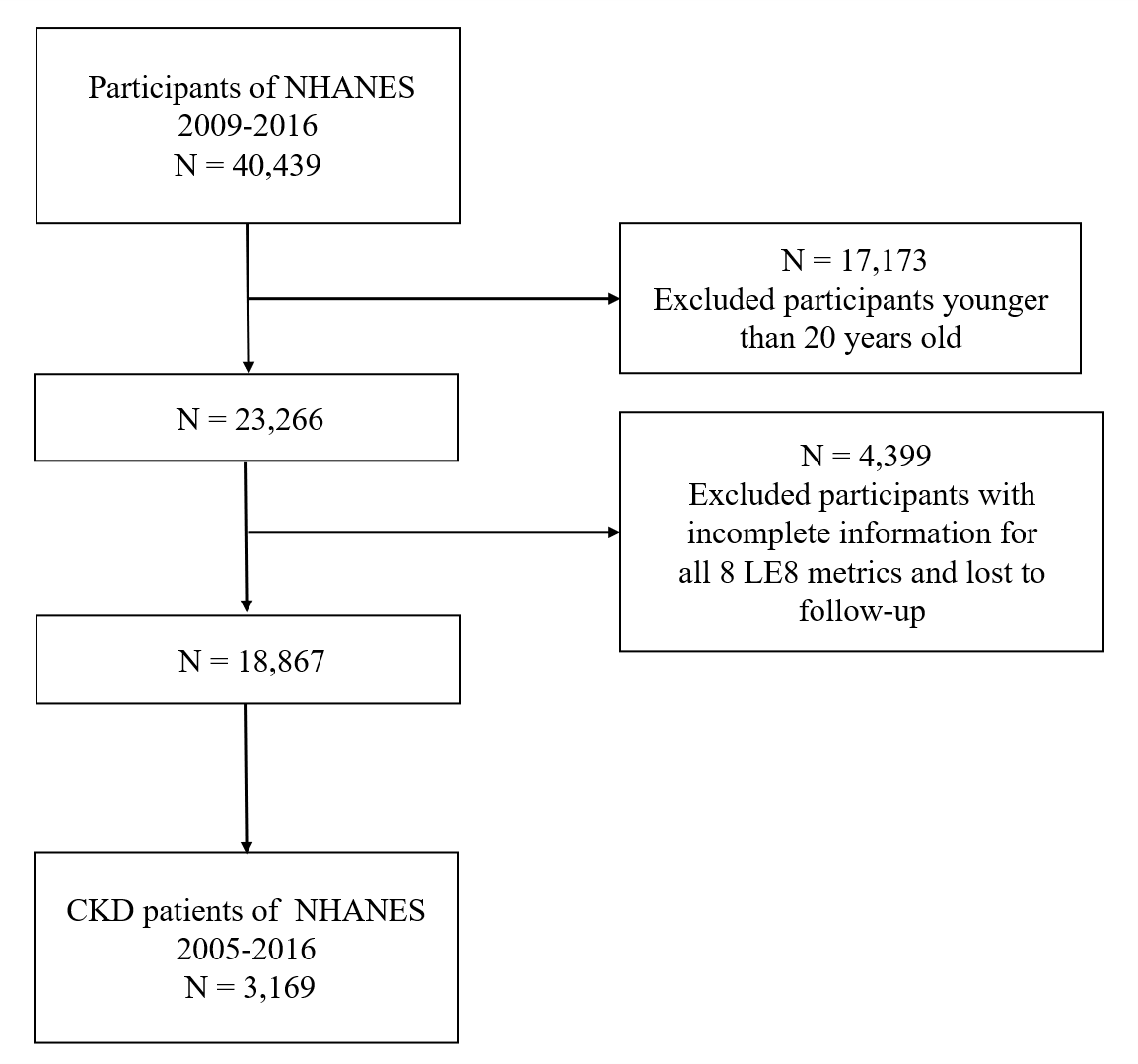
**Additional Figure 1.** Flow chart of the screening process for the selection of the study population.

Abbreviations: NHANES, national health and nutrition examination surveys; LE8, Life’s Essential 8.

**Additional** **Table 1.** Definition and scoring approach for the American Heart Association’s Life’s Essential 8 score.

| Domain | CVH Metric | Measurement | Quantification and Scoring of CVH Metric |
| --- | --- | --- | --- |
| Health Behaviors | Diet | Healthy Eating Index-2015 diet score percentile | Quantiles of DASH-style diet adherence  **Scoring (Population):**  Points Quantile  100 ≥95^th^ percentile (top/ideal diet)  80 75^th^ – 94^th^ percentile  50 50^th^ – 74^th^ percentile  25 25^th^ – 49^th^ percentile  0 1^st^ – 24^th^ percentile (bottom/least ideal quartile) |
|  | Physical activity | Self-reported minutes of moderate or vigorous physical activity per week | **Metric:** Minutes of moderate (or greater) intensity activity per week  **Scoring:**  Points Minutes  100 ≥150  90 120 – 149  80 90 – 119  60 60 – 89  40 30 – 59  20 1 – 29  0 0 |
|  | Nicotine exposure | Self-reported use of cigarettes or inhaled nicotine- delivery system | **Metric:** Combustible tobacco use and/or inhaled NDS use; or secondhand smoke exposure  **Scoring:**  Points Status  100 Never smoker  75 Former smoker, quit ≥5 yrs  50 Former smoker, quit 1 - <5 yrs  25 Former smoker, quit <1 year, or currently using inhaled NDS  0 Current smoker  Subtract 20 points (unless score is 0) for living with active indoor smoker in home |
|  | Sleep health | Self-reported average hours of sleep per night | **Metric:** Average hours of sleep per night  **Scoring:**  Points Level  100 7 – <9  90 9 – <10  70 6 – <7  40 5 – <6 or ≥10  20 4 – <5  0 <4 |
| Health Factors | Body mass index | Body weight (kg) divided by height squared (m^2^) | **Metric:** Body mass index (kg/m^2^)  **Scoring:** Points Level 100 <25  70 25.0 – 29.9  30 30.0 – 34.9  15 35.0 – 39.9  0 ≥40.0 |
|  | Blood lipids | Plasma total and HDL-cholesterol with calculation of non-HDL-cholesterol | **Metric:** Non-HDL-cholesterol (mg/dL)  **Scoring:**  Points Level  100 <130  60 130 – 159  40 160 – 189  20 190 – 219  0 ≥220  If drug-treated level, subtract 20 points |
|  | Blood glucose | Fasting blood glucose or casual hemoglobin A1c | **Metric:** Fasting blood glucose (mg/dL) or Hemoglobin A1c (%)  **Scoring:**  Points Level  100 No history of diabetes and FBG <100 (or HbA1c < 5.7)  60 No diabetes and FBG 100 – 125 (or HbA1c 5.7-6.4) (Pre-diabetes)  40 Diabetes with HbA1c <7.0  30 Diabetes with HbA1c 7.0 – 7.9  20 Diabetes with HbA1c 8.0 – 8.9  10 Diabetes with Hb A1c 9.0 – 9.9  0 Diabetes with HbA1c ≥10.0 |
|  | Blood pressure | Appropriately measured systolic and diastolic blood pressure | **Metric:** Systolic and diastolic blood pressure (mm Hg)  **Scoring:**  Points Level  100 <120/<80 (Optimal)  75 120-129/<80 (Elevated)  50 130-139 or 80-89 (Stage I HTN)  25 140-159 or 90-99  0 ≥160 or ≥100  Subtract 20 points if treated level |

**Reference**

1. Lloyd-Jones DM, Allen NB, Anderson CAM, et al. Life's Essential 8: Updating and Enhancing the American Heart Association's Construct of Cardiovascular Health: A Presidential Advisory From the American Heart Association. *Circulation*. Aug 2 2022;146(5):e18-e43.
2. Lloyd-Jones DM, Ning H, Labarthe D, et al. Status of Cardiovascular Health in US Adults and Children Using the American Heart Association's New "Life's Essential 8" Metrics: Prevalence Estimates From the National Health and Nutrition Examination Survey (NHANES), 2013 Through 2018. *Circulation*. Sep 13 2022;146(11):822-835.

**Additional** **Table 2.** Healthy Eating Index-2015 Components & Scoring Standards^1^

| Component | Maximum points | Standard for maximum score | Standard for minimum score of zero |
| --- | --- | --- | --- |
| *Adequacy* | | | |
| Total Fruits[^2^](https://epi.grants.cancer.gov/hei/developing.html#f2) | 5 | ≥0.8 cup equiv. per 1,000 kcal | No Fruit |
| Whole Fruits[^3^](https://epi.grants.cancer.gov/hei/developing.html#f3) | 5 | ≥0.4 cup equiv. per 1,000 kcal | No Whole Fruit |
| Total Vegetables[^4^](https://epi.grants.cancer.gov/hei/developing.html#f4) | 5 | ≥1.1 cup equiv. per 1,000 kcal | No Vegetables |
| Greens and Beans[^4^](https://epi.grants.cancer.gov/hei/developing.html#f4) | 5 | ≥0.2 cup equiv. per 1,000 kcal | No Dark Green Vegetables or Legumes |
| Whole Grains | 10 | ≥1.5 oz equiv. per 1,000 kcal | No Whole Grains |
| Dairy[^5^](https://epi.grants.cancer.gov/hei/developing.html#f5) | 10 | ≥1.3 cup equiv. per 1,000 kcal | No Dairy |
| Total Protein Foods[^6^](https://epi.grants.cancer.gov/hei/developing.html#f6) | 5 | ≥2.5 oz equiv. per 1,000 kcal | No Protein Foods |
| Seafood and Plant Proteins[^6^](https://epi.grants.cancer.gov/hei/developing.html#f6)^,^[^7^](https://epi.grants.cancer.gov/hei/developing.html#f7) | 5 | ≥0.8 oz equiv. per 1,000 kcal | No Seafood or Plant Proteins |
| Fatty Acids[^8^](https://epi.grants.cancer.gov/hei/developing.html#f8) | 10 | (PUFAs + MUFAs)/SFAs ≥2.5 | (PUFAs + MUFAs)/SFAs ≤1.2 |
| *Moderation* | | | |
| Refined Grains | 10 | ≤1.8 oz equiv. per 1,000 kcal | ≥4.3 oz equiv. per 1,000 kcal |
| Sodium | 10 | ≤1.1 gram per 1,000 kcal | ≥2.0 grams per 1,000 kcal |
| Added Sugars | 10 | ≤6.5% of energy | ≥26% of energy |
| Saturated Fats | 10 | ≤8% of energy | ≥16% of energy |

**(1)** Intakes between the minimum and maximum standards are scored proportionately.

**(2)** Includes 100% fruit juice.

**(3)** Includes all forms except juice.

**(4)** Includes legumes (beans and peas).

**(5)** Includes all milk products, such as fluid milk, yogurt, and cheese, and fortified soy beverages.

**(6)** Includes legumes (beans and peas).

**(7)** Includes seafood, nuts, seeds, soy products (other than beverages), and legumes (beans and peas).

**(8)** Ratio of poly- and monounsaturated fatty acids (PUFAs and MUFAs) to saturated fatty acids (SFAs).

*Adequacy components* represent the food groups, subgroups, and dietary elements that are encouraged. For these components, higher scores reflect higher intakes, because higher intakes are desirable.

*Moderation components* represent the food groups and dietary elements for which there are recommended limits to consumption. For moderation components, higher scores reflect lower intakes, because lower intakes are more desirable.

**Reference**

1. Krebs-Smith SM, Pannucci TE, Subar AF, et al. Update of the Healthy Eating Index: HEI-2015. J Acad Nutr Diet. Sep 2018;118(9):1591-1602.

2. National Cancer Institute. HEI Scoring Algorithm. Accessed August, 2022. https://epi.grants.cancer.gov/hei/hei-scoring-method.ht.

**Additional Table 3.** Clinical characteristic of the study population by cardiovascular health (CVH) status.

|  | Overall^*^  (n = 3169) | Low CVH  (n = 898) | Moderate CVH  (n = 2042) | High CVH  (n = 229) | *P* Value |
| --- | --- | --- | --- | --- | --- |
| Weighted n | 26857095 | 6551503 | 17473023 | 2832569 |  |
| Age (year) ^†^ | 66.0 (25.0) | 65.0 (20.0) | 68.0 (26.0) | 43.0 (42.0) | <0.01 |
| Female (%) ^‡^ | 1671 (52.7) | 475 (52.9) | 1047 (51.3) | 149 (65.1) | <0.01 |
| Race (%) ^‡^ |  |  |  |  | <0.01 |
| Mexican American | 417 (13.2) | 145 (16.1) | 247 (12.1) | 25 (10.9) |  |
| Other Hispanic | 291 (9.2) | 87 (9.7) | 182 (8.9) | 22 (9.6) |  |
| NH White | 1470 (46.4) | 371 (41.3) | 981 (48.0) | 118 (51.5) |  |
| NH Black | 714 (22.5) | 242 (26.9) | 444 (21.7) | 28 (12.2) |  |
| Other Race | 277 (8.7) | 53 (5.9) | 373 (20.0) | 44 (20.9) |  |
| Education level (%) ^‡^ |  |  |  |  | <0.01 |
| High school or less | 1708 (53.9) | 569 (63.4) | 1067 (52.3) | 72 (31.4) |  |
| Some college or AA | 884 (27.9) | 243 (27.1) | 590 (28.9) | 51 (22.3) |  |
| College graduate or above | 574 (18.1) | 86 (9.6) | 382 (18.7) | 106 (46.3) |  |
| Marital Status (%) ^‡^ |  |  |  |  | 0.15 |
| Coupled | 1734 (54.8) | 471 (52.4) | 1128 (55.3) | 135 (59.0) |  |
| Single or separated | 1433 (45.2) | 427 (47.6) | 912 (44.7) | 94 (41.0) |  |
| Poverty ratio (%) ^‡^ |  |  |  |  | <0.01 |
| < 1.0 | 686 (23.6) | 269 (32.4) | 373 (20.0) | 44 (20.9) |  |
| ≥ 1.0 | 2221 (76.4) | 561 (67.6) | 1493 (80.0) | 167 (79.1) |  |
| Obesity status (%) ^‡^ |  |  |  |  | <0.01 |
| Normal | 750 (23.7) | 78 (8.7) | 521 (25.5) | 151 (65.9) |  |
| Overweight | 989 (31.2) | 178 (19.8) | 746 (36.5) | 65 (28.4) |  |
| Obesity | 1430 (45.1) | 642 (71.5) | 775 (38.0) | 13 (5.7) |  |
| Uric acid (mg/dL) | 5.9 (2.2) | 6.2 (2.4) | 5.9 (2.2) | 5.1 (2.0) | <0.01 |
| Creatinine (μmol/L) | 92.8 (46.0) | 93.7 (47.7) | 94.6 (46.0) | 78.68 (40.7) | <0.01 |
| eGFR (mL/min/1.73 m²) | 61.32 (44.87) | 63.02 (44.56) | 59.53 (42.89) | 89.74 (57.32) | <0.01 |
| UACR (mg/g) | 44.5 (94.8) | 57.2 (133.7) | 41.1 (84.2) | 41.88 (49.9) | <0.01 |
| CKD stages 3 (%) ^‡^ | 1371 (43.3) | 359 (40.0) | 945 (46.3) | 67 (29.4) | <0.01 |
| CKD stages 4-5 (%) ^‡^ | 183 (5.8) | 61 (6.8) | 118 (5.8) | 4 (1.7) | 0.014 |
| CKD stages 3-5 (%) ^‡^ | 1554 (49.0) | 420 (46.8) | 1063 (52.1) | 71 (31.0) | <0.01 |
| LE8 scores (out of 100 possible points) ^†^ | 57.5 (19.4) | 42.5 (8.8) | 61.9 (13.1) | 85.0 (6.9) | <0.01 |
| Health behaviors score ^†^ | 62.5 (26.3) | 43.8 (20.0) | 67.5 (21.3) | 87.5 (15.0) | <0.01 |
| Health factors score ^†^ | 53.8 (26.3) | 37.5 (18.8) | 57.5 (21.3) | 87.5 (23.8s) | <0.01 |

Data are number of subjects (percentage) or medians (interquartile ranges).

UACR: Urine albumin creatinine ratio.

* Numbers of each stratum may not add up to the total population due to missing data.

† Kruskal Wallis test was used to compare the median values among participants in different groups.

‡ Chi-square test was used to compare the percentage among participants in different groups.

**Additional Figure 2.** Subgroup analysis of the association of the Life’s Essential 8 scores and all-cause mortality of chronic kidney disease patients.


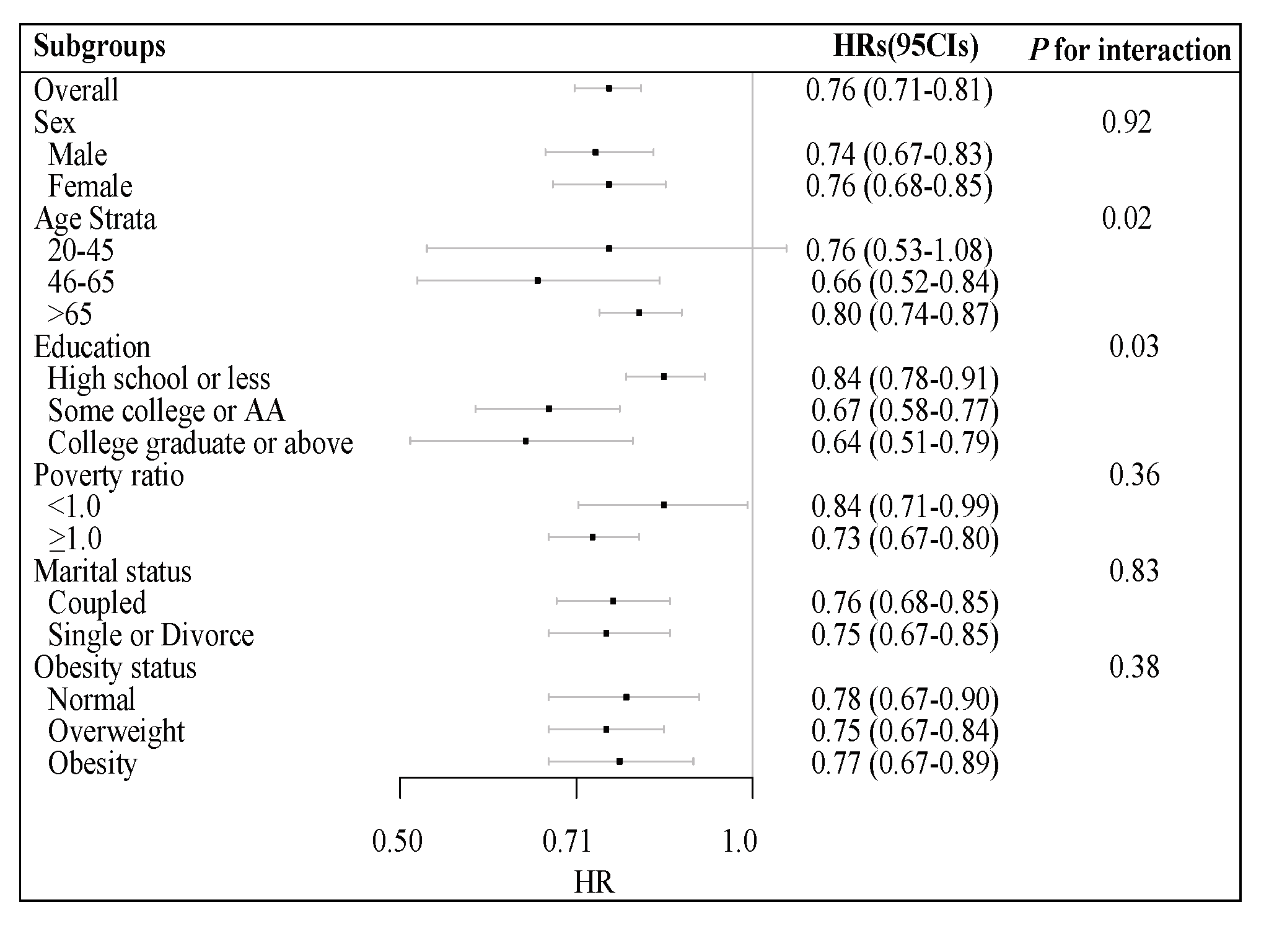


HRs were calculated as per 10 scores increase in LE8 score. Each stratification was adjusted for age, sex, race, obesity status, uric acid, poverty ratio (as a binary variable), education level and marital status.

**Additional Table 4.** Characteristics of the matched study population.

|  | Survival group  (n = 629) | Mortality group  (n = 629) | SMD | *P* value |
| --- | --- | --- | --- | --- |
| Age (years) | 71.70 (9.87) | 71.78 (10.39) | 0.008 | 0.889 |
| Female (%) | 322 (51.2) | 327 (52.0) | 0.016 | 0.821 |
| Race/Ethnicity (%) |  |  | 0.099 | 0.541 |
| Mexican American | 67 (10.7) | 61 (9.7) |  |  |
| Other Hispanic | 47 (7.5) | 38 (6.0) |  |  |
| NH White | 363 (57.7) | 370 (58.8) |  |  |
| NH Black | 129 (20.5) | 127 (20.2) |  |  |
| Other Race | 23 (3.7) | 33 (5.2) |  |  |
| Education (%) |  |  | 0.050 | 0.678 |
| High school or less | 384 (61.0) | 372 (59.1) |  |  |
| Some college or AA | 163 (25.9) | 165 (26.2) |  |  |
| College graduate or above | 82 (13.0) | 92 (14.6) |  |  |
| Single or Divorce (%) | 314 (49.9) | 323 (51.4) | 0.029 | 0.652 |
| Poverty ratio < 1.0 (%) | 125 (19.9) | 141 (22.4) | 0.062 | 0.300 |
| Obesity status (%) |  |  | 0.050 | 0.673 |
| Normal | 167 (26.6) | 155 (24.6) |  |  |
| Overweight | 202 (32.1) | 214 (34.0) |  |  |
| Obesity | 260 (41.3) | 260 (41.3) |  |  |
| Uric acid (mg/dL) | 6.32 (1.44) | 6.29 (1.71) | 0.020 | 0.725 |

SMD, standardized mean difference.

**Additional Figure 3.** Distribution of propensity score before and after matching.
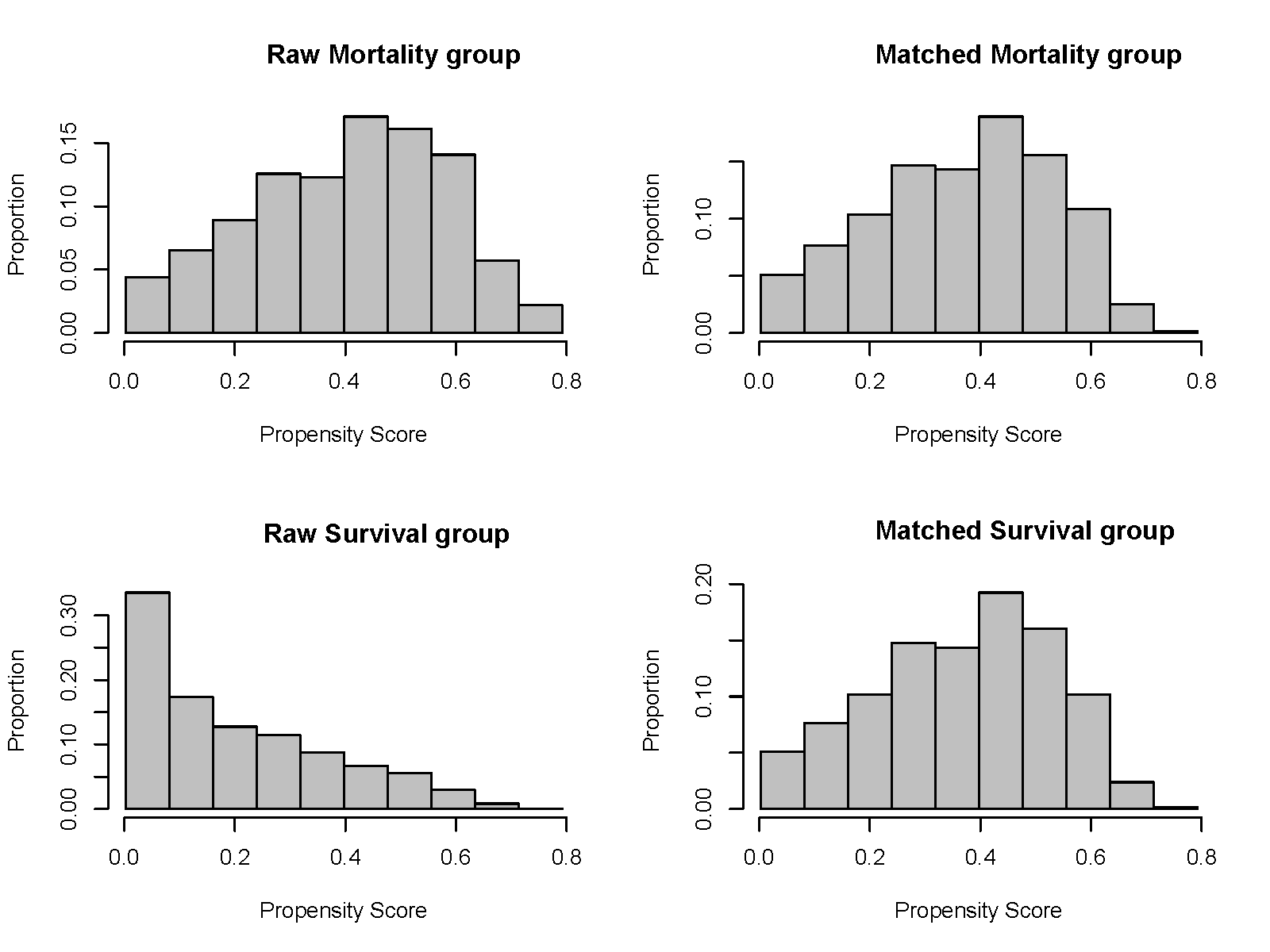


Matching for age, sex, race, obesity status, uric acid, education levels, poverty status and marital status. with caliper = 0.02.
